# Incidence of Permanent Pacemaker Implantation Following Tricuspid Valve Surgeries: A Systematic Review and Meta-Analysis

**DOI:** 10.1177/15569845261455899

**Published:** 2026-06-16

**Authors:** Seyed Shervin Shafiei, Saeid Hosseini, Samuel Heuts, Abolfazl Hosseinnataj, Peyman Sardari Nia

**Affiliations:** 1 Cardiovascular Research Institute Maastricht (CARIM), Maastricht University, The Netherlands; 2 Heart Valve Diseases Research Center, Rajaie Cardiovascular Institute, Tehran, Iran; 3 Cardiothoracic Surgery, Maastricht University Medical Center (MUMC+), The Netherlands; 4 Department of Biostatistics and Epidemiology, Faculty of Health, Mazandaran University of Medical Sciences, Sari, Iran

**Keywords:** tricuspid valve, pacemaker, systematic review, meta-analysis

## Abstract

**Objective:**

Tricuspid valve (TV) surgery is increasingly performed. Still, the risk of permanent pacemaker (PPM) implantation following TV surgery is a serious concern. Therefore, the present systematic review and meta-analysis is aimed to determine the incidence of PPM implantation following TV surgeries in various surgical settings.

**Methods:**

This study was preregistered in PROSPERO (CRD42024585421). A methodical search was performed across various international electronic databases. Random-effects models were used to illustrate the aggregated incidence of PPM implantation following different types of TV surgeries. Meta-regression and subgroup analysis were applied to evaluate the statistically significant effect of modifiers.

**Results:**

Twelve studies were included; a total of 18,437 patients underwent TV surgery. The incidence of PPM implantation following overall TV surgery was 12.1% (*P* < 0.001), isolated TV surgery was 11.1% (*P* < 0.001), TV + mitral valve (MV) surgery was 12.3% (*P* < 0.001), TV + aortic valve (AV) surgery was 10.9% (*P* = 0.001), and TV + MV + AV surgery was 17.3% (*P* < 0.001).

**Conclusions:**

PPM implantation is a common complication that can arise after TV surgery, especially when multiple valves are involved. Although substantial variability across studies makes it difficult to pinpoint exact risks, the overall message is clear: there is a need to focus on preoperative patient counseling and develop standardized research to improve our ability to predict and prevent these complications.

## Graphical abstract

**Graphical Abstract:**
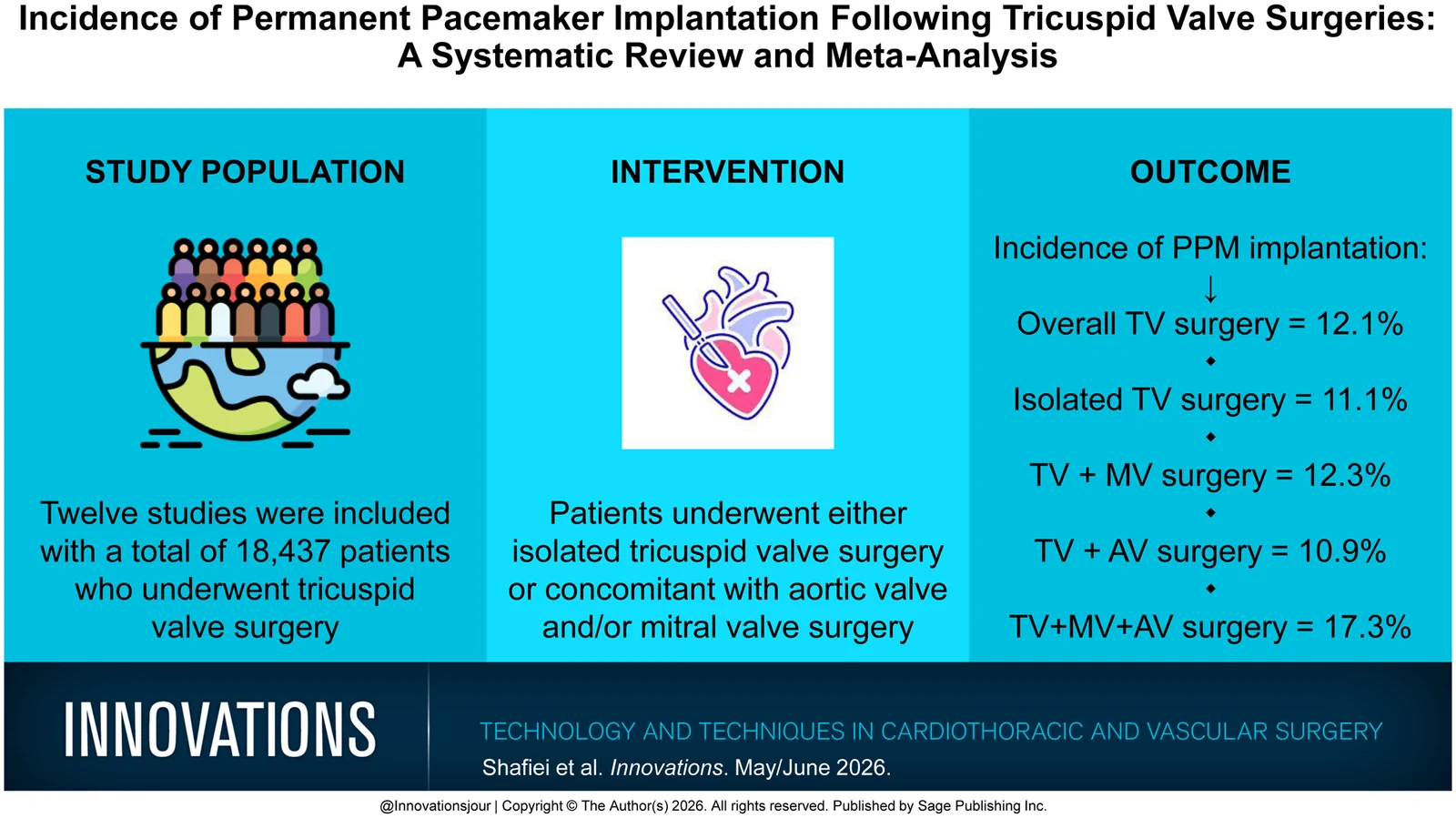
This is a visual representation of the abstract.

**Central Message**

PPM implantation occurs in ~12% of patients after TV surgery, regardless of isolated or concomitant procedures, highlighting the importance of surgical planning and patient counseling regarding conduction system risk.

## Introduction

Tricuspid valve (TV) surgery is an important intervention for patients with significant TV disease, particularly those with severe regurgitation or stenosis.^1,2^ If left untreated, severe tricuspid regurgitation can substantially impair quality of life, worsen right ventricular function, and increase both morbidity and mortality.^3,4^ Surgical or transcatheter interventions on the TV aim to restore valve competence and prevent progressive right heart failure.^5^

Despite advancements in operative techniques and perioperative care, postoperative conduction disturbances remain a notable concern following TV surgery.^6^ The anatomical proximity of the TV annulus to the atrioventricular conduction system predisposes patients to conduction injury during surgical manipulation.^7,8^ Such injury can lead to varying degrees of atrioventricular block, sometimes necessitating permanent pacemaker (PPM) implantation to maintain hemodynamic stability.^9,10^ The risk is particularly elevated when TV surgery is performed concomitantly with other valve procedures, such as mitral valve (MV) or aortic valve (AV) repair or replacement.^11,12^

Previous studies have reported highly variable rates of PPM implantation after TV surgery, with the incidence differing across surgical types, patient populations, and institutional practices.^11–14^ Factors such as operative duration, type of valve intervention (repair vs replacement), and preexisting comorbidities (e.g., ischemic heart disease, diabetes, or heart failure) may further influence the likelihood of PPM implantation.^12,14,15^ However, the true pooled incidence and the influence of these risk factors remain uncertain due to inconsistencies in study design and reporting.

To date, no comprehensive quantitative synthesis has evaluated the incidence of PPM implantation across different categories of TV surgery, including isolated and multivalve procedures. Therefore, the present systematic review and meta-analysis aims to determine the cumulative incidence of PPM implantation following various types of TV surgeries and identify clinical and procedural factors associated with increased pacing risk. By consolidating available evidence, this study seeks to provide a clearer understanding of the burden of conduction disturbances after TV surgery and to support improved perioperative planning and patient counseling.

## Methods

This systematic review and meta-analysis followed the 2020 PRISMA checklist for Preferred Reporting Items.^16^ This study was registered in the International Prospective Register of Systematic Reviews (PROSPERO) with code CRD42024585421 (dated September 24, 2024).

### Search Strategy

A comprehensive and systematic search was made through several international electronic databases, including Scopus, PubMed, Web of Science, and Persian databases such as Iranmedex and Scientific Information Database (SID). A comprehensive literature search was performed using the keywords from MeSH: “Incidence,” “tricuspid valve,” and “artificial pacemaker,” covering the period from the earliest records available up to August 1, 2024. The search for records in PubMed, for example, was based on the following query: (“Incidence”[MeSH Terms] OR incidence [Text Word]) AND (“Pacemaker, Artificial”[MeSH Terms] OR “permanent pacemaker”[Text Word]) AND (“Tricuspid Valve”[MeSH Terms] OR “tricuspid valve surgery”[Text Word]). Searching and screening were carried out by 2 independent researchers. We included only English and Persian studies that were in peer-reviewed journal articles, clinical trials, and observational studies. Gray literature, such as expert opinions, conference presentations, theses, research reports, and ongoing research, were excluded due to the nature of rigor we wanted to maintain in the study. Duplicate studies identified across the databases were removed. The final selection of studies was based on their relevance to the research question and their methodological quality.

### Inclusion and Exclusion Criteria

Eligible studies included those reporting TV surgery as an isolated procedure or in combination with MV and/or AV surgery. Studies primarily evaluating surgical ablation for atrial fibrillation were excluded; however, studies in which a subset of patients underwent concomitant ablation for atrial fibrillation were eligible, as ablation status was inconsistently reported and not extractable for subgroup analysis. Also, our analysis excluded reviews, case studies, conference proceedings, letters to the editor, legal documents, and qualitative research.

### Study Selection

EndNote 21 (Clarivate, London, UK) was used for data management in this systematic review. There were numerous steps in the selection process based on inclusion and exclusion criteria. (1) The first step was to look at the titles and abstracts of the research. (2) Both computerized and manual methods were used to find duplicates. (3) Full-text publications were evaluated to determine final decisions regarding inclusion or exclusion. If the initial 2 researchers could not agree on something during the selection process, a third researcher was called in to settle the disagreement. (4) As a last step, a full check of the references was done to make sure that no important information was missed.

### Data Extraction and Quality Assessment

In this review, the researchers gathered multiple data points, such as the first author name, publication year, study location, total sample size, male-to-female ratio, age, type of TV surgery, sample size segmented by surgery type, and related risk factors. Information on the etiology of TV disease (functional vs organic) was extracted when reported. However, most studies did not specify the underlying pathology, limiting our ability to perform stratified analyses based on disease type. We evaluated the risk of bias using the ROBINS-I tool, specifically designed for observational cohort studies.^17^ This tool looks at 7 key areas including confounding, how participants are selected, how interventions are classified, any deviations from the planned interventions, missing data, how outcomes are measured, and selective reporting. Each study was rated as having low, moderate, or serious risk of bias. For the 1 randomized controlled trial, we used the Cochrane RoB-2 tool.^18^ Two researchers independently extracted data and assessed the risk of bias, and any differences were resolved through discussion and agreement.

### Statistical Analysis

For the analysis, we used the Comprehensive Meta-Analysis Software program, version 4 (Biostat, Inc., Englewood, NJ, USA). The weight assigned to each study was determined by its inverse variance. Heterogeneity among the studies was assessed and visualized using a forest plot. We compiled the sample size and frequency of PPM implantation for each study, which was then used to calculate the overall effect size. The I^2^ statistic was used to measure the level of heterogeneity. Values from 25% to 50% were considered low heterogeneity, 51% to 75% were moderate heterogeneity, and >75% were high heterogeneity. We anticipated substantial clinical and methodological heterogeneity due to the projected variability in patient populations, surgical procedures, and institutional policies among trials. Therefore, a meta-analysis was still considered appropriate to provide an overall quantitative synthesis of the available evidence, in line with the recommendations of the Cochrane Handbook and the PRISMA guidelines, provided that the results are interpreted with caution.^16,19^ A random-effects model was chosen a priori, as it accounts for variability between studies and does not assume a single common effect size. Subgroup analyses were performed to explore the odds ratio (OR) of PPM implantation based on study-specific variables, such as gender and incidence differences between countries. An additional subgroup analysis comparing TV repair versus replacement was performed when study-level data were available. In addition, we measured the incidence of PPM implantation across different risk factors, including ischemic heart disease, diabetes, hypertension, and heart failure. Meta-regression was applied to evaluate statistically significant effect modifiers.

#### Sensitivity analysis

A sensitivity analysis was performed to evaluate the impact of excluding each study on the overall incidence of PPM implantation following TV surgeries (leave-one-out analysis).

#### Publication bias

To evaluate the potential for publication bias, we employed the Egger test in conjunction with a funnel plot analysis, for which a *P* value of <0.05 denoted the presence of statistically significant publication bias.

## Results

### Study Selection

**Fig. 1.**
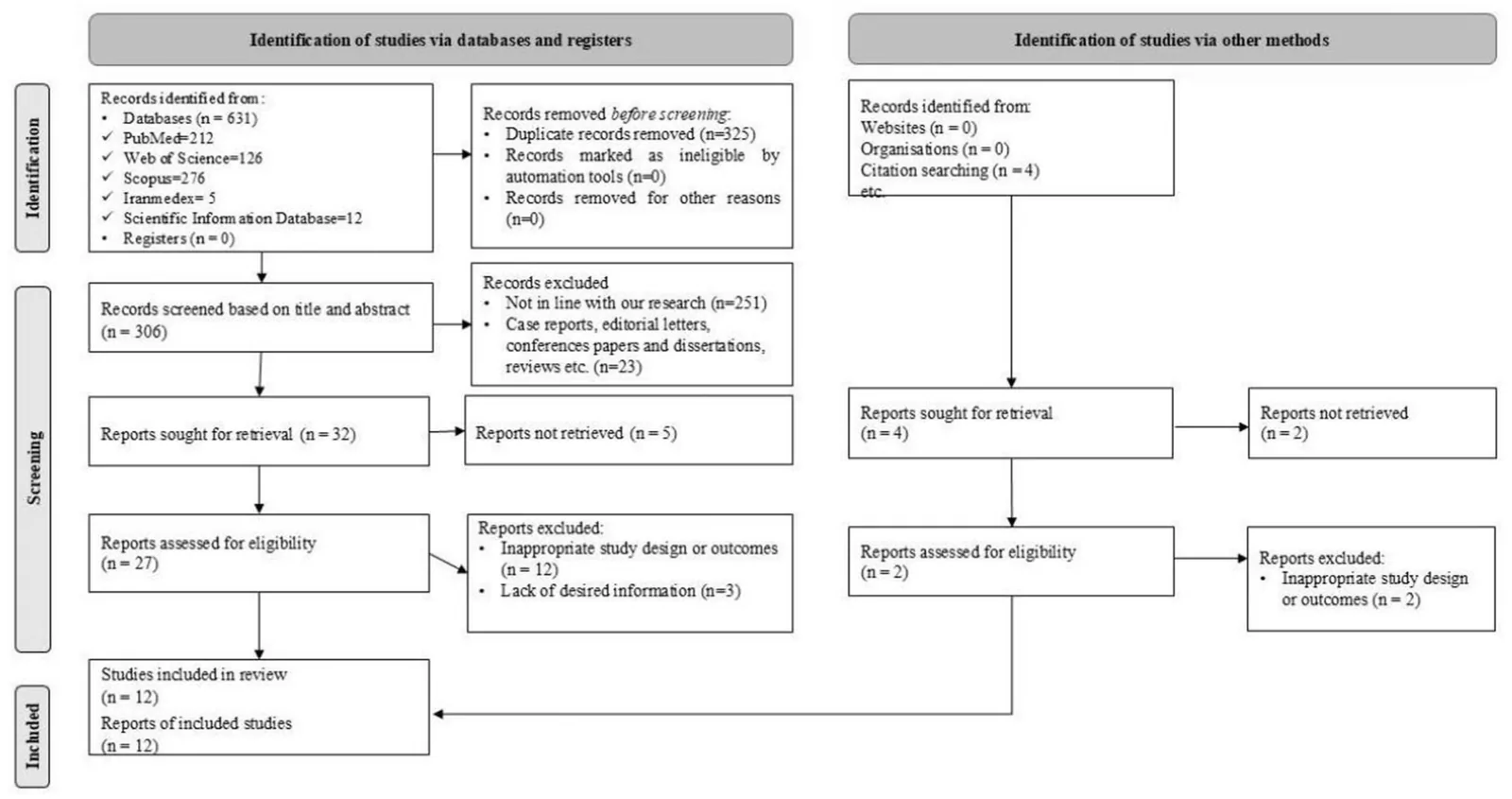
Flow diagram of the study selection process.

The initial database search for this systematic review and meta-analysis identified a total of 631 studies. After removing duplicates, 306 studies remained. A thorough screening of titles and abstracts led to the exclusion of 251 studies that did not meet the inclusion criteria. An additional 23 studies were excluded due to containing case reports, editorial letters, conference abstracts, dissertations, reviews, or other nonresearch-related content. Following a detailed full-text review of 27 studies, 12 were excluded due to irrelevant outcomes, and 3 were excluded for lacking sufficient data. Ultimately, 12 studies were included in the final analysis (Fig. 1).

A total of 18,437 subjects across 12 studies underwent TV surgery (Table 1).^6,11–15,20–25^ The average age of the participants was 66.34 years (standard deviation = 2.11 years), with 39.4% being male patients (95% confidence interval [CI]: 27.2% to 53.0%). Of the total surgeries, 4,603 involved isolated TV surgery, 10,878 were concomitant surgeries on the TV and MV, 1,305 involved both the TV and AV, and 1,651 were performed on the TV, MV, and AV.

**Table 1.** Basic Characteristics of the Included Studies in this Systematic Review and Meta-Analysis.

First author, year	Location	Total sample size	M/F, *n*	Mean age, years	TV surgery	TV replace	TV repair
Cooper et al., 1995^22^	UK	45	11/34	60.4 (SD = 1.5)	Only TV (*n* = 3)	3	0
					TV+MV (*n* = 36)	36	0
					TV+MV+AV (*n* = 6)	6	0
Mar et al., 2017^13^	USA	237	82/155	63.10 (SD = 15.20)	Only TV (*n* = 32)	NR	NR
					TV+MV (*n* = 100)	NR	NR
					TV+AV (*n* = 7)	NR	NR
					TV+MV+AV (*n* = 22)	NR	NR
Kho et al., 2020^6^	UK	403	287/116	66 (SD = 10)	Only TV (*n* = 16)	NR	NR
					TV+MV (*n* = 12)	NR	NR
Chi et al., 2021^21^	Taiwan	24,014	13,968/10,046	59.82	Only TV (*n* = 744)	NR	NR
					TV+MV (*n* = 1,743)	NR	NR
					TV+AV (*n* = 176)	NR	NR
					TV+MV+AV (*n* = 442)	NR	NR
Gammie et al., 2022^25^	USA, Canada, Germany	401	N/A	66.6 (SD = 10.7)	TV+MV (*n* = 198)	0	198
Ragnarsson et al., 2023^14^	Sweden	1,502	974/528	67.8 (SD = 12.2)	Only TV (*n* = 152)	0	152
					TV+MV (*n* = 1,127)	0	1,127
					TV+AV (*n* = 239)	0	239
Ailawadi et al., 2024^24^	USA, Canada, Germany	401	NR	NR	TV+MV (*n* = 188)	0	188
Brescia et al., 2024^20^	USA	1,738	NR	NR	TV+MV (*n* = 417)	0	417
Fu et al., 2024^11^	USA	1,346	684/662	66.00	Only TV (*n* = 214)	79	135
					TV+MV (*n* = 868)	19	849
					TV+AV (*n* = 74)	9	65
					TV+MV+AV (*n* = 130)	3	127
Faerber et al., 2024^15^	Multicountry	7,513	1,004/1,216	71.00	TV+MV (*n* = 1,110)	0	1,110
Kassab et al., 2024^12^	USA	13,294	5,465/7,829	71.7 (SD = 11.0)	Only TV (*n* = 3442)	2,123	1,319
					TV+MV (*n* = 4,852)	811	4,041
					TV+AV (*n* = 809)	182	627
					TV+MV+AV (*n* = 1,052)	190	862
Olsthoorn et al., 2024^23^	Netherlands	1,060	143/84	71.00	TV+MV (*n* = 227)	NR	NR

Abbreviations: AV, aortic valve; F, female; M, male; MV, mitral valve; NR, not reported; SD, standard deviation; TV, tricuspid valve.

### Methodological Quality Assessment of Eligible Studies

In accordance with the ROBINS-I framework, several observational studies were judged to have a moderate risk of bias (Supplemental Fig. 1).^11,12,15,21,23,24^ This was mainly due to factors such as confounding, deviations from the intended interventions, and the management of missing data. By contrast, a number of studies showed a low overall risk of bias.^6,13,14,20^ However, 1 study raised serious concerns, particularly regarding confounding and how participants were selected, leading to a serious risk of bias.^22^ The randomized controlled trial, assessed using the RoB-2 tool, indicated a low risk of bias across various domains, with just a few minor issues related to deviations from the planned interventions.^25^

### Incidence of PPM Implantation Following Different TV Surgeries

**Fig. 2.**
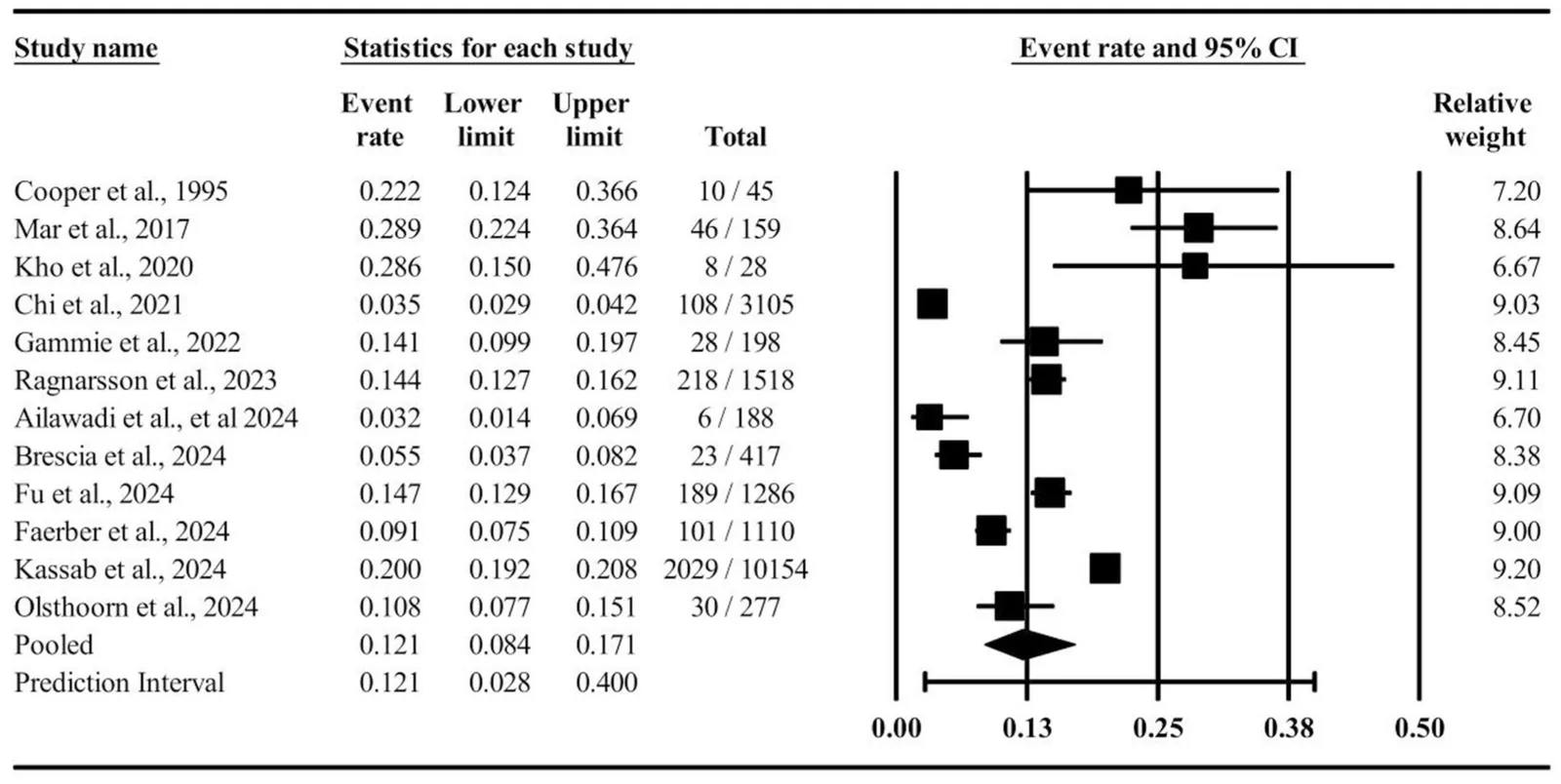
Forest plot incidence of permanent pacemaker implantation following overall tricuspid valve surgeries. CI, confidence interval.

**Fig. 3.**
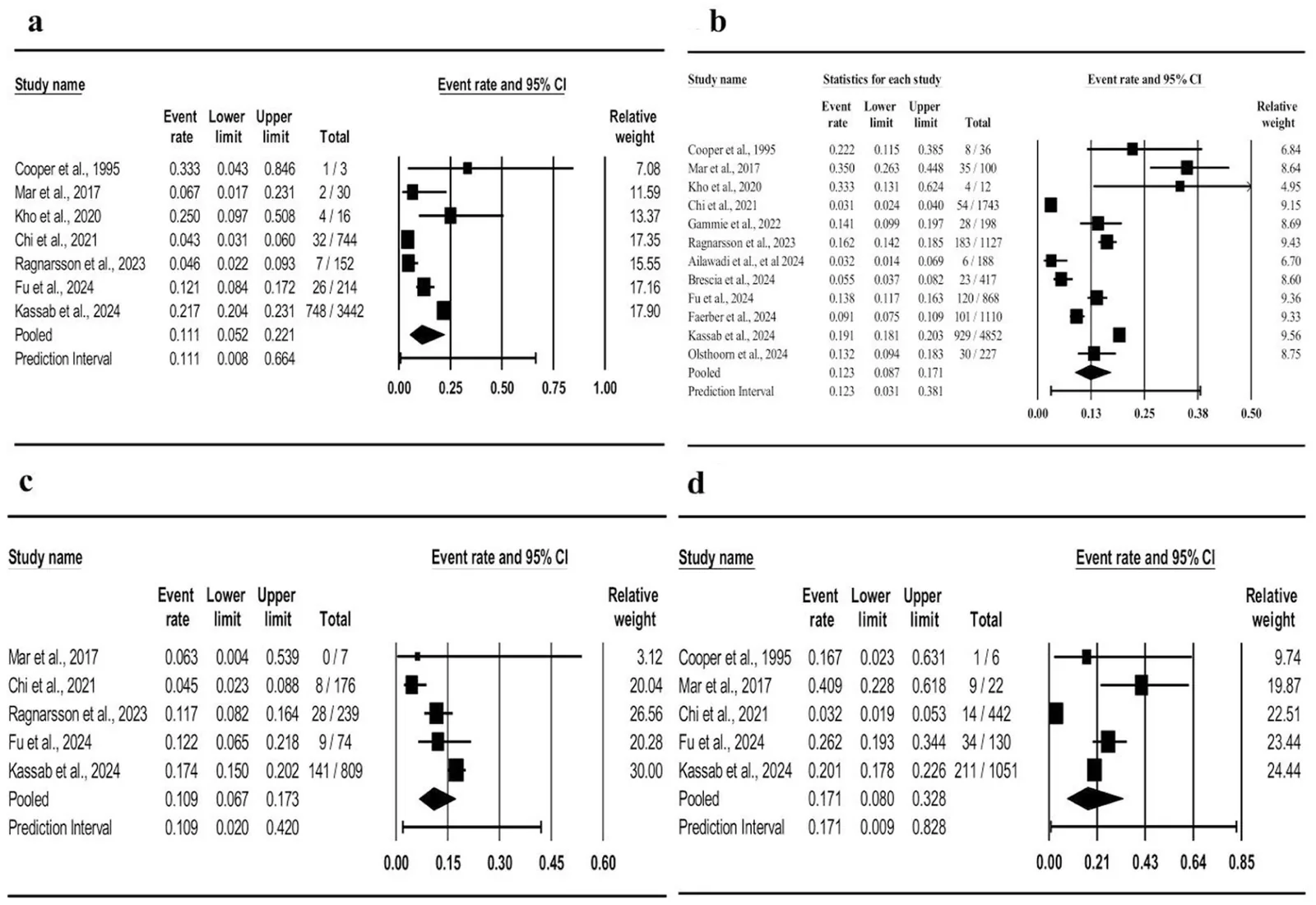
Forest plot incidence of permanent pacemaker implantation following (a) TV surgery, (b) TV+MV surgery, (c) TV+AV surgery, and (d) TV+MV+AV surgery. AV, aortic valve; CI, confidence interval; MV, mitral valve; TV, tricuspid valve.

Study-level PPM implantation incidences and related factors across the included studies are summarized in Table 2. The incidence of PPM implantation following overall TV surgeries was 12.1% (95% CI: 8.4% to 17.1%, I^2^ = 97.88%, *P* < 0.001; Fig. 2), isolated TV surgery was 11.1% (95% CI: 5.2% to 22.1%, I^2^ = 95.23%, *P* < 0.001; Fig. 3a), TV+MV surgery was 12.3% (95% CI: 8.7% to 17.1%, I^2^ = 96.58%, *P* < 0.001; Fig. 3b), TV+AV surgery was 10.9% (95% CI: 6.7% to 17.3%, I^2^ = 79.74%, *P* = 0.001; Fig. 3c), and TV+MV+AV surgery was 17.3% (95% CI: 8.0% to 32.8%, I^2^ = 93.74%, *P* < 0.001; Fig. 3d).

**Table 2.** PPM Implantation Incidence and Related Factors.

First author, year	Only TV	TV+MV	TV+AV	TV+MV+AV	TV replace	TV repair	Male	Female	Ischemic heart disease	Diabetes	Hypertension	Heart failure
Cooper et al., 1995^22^	1/3	8/36	—	1/6	10/45	—	—	—	1/1	—	—	—
Mar et al., 2017^13^	2/30	35/100	0/7	9/22	—	—	19/82	46/155	—	14/52	49/164	—
Kho et al., 2020^6^	4/16	4/12	—	—	—	—	—	—	—	—	—	—
Chi et al., 2021^21^	32/744	54/1,743	8/176	14/442	—	—	—	—	—	—	—	—
Gammie et al., 2022^25^	—	28/198	—	—	—	28/198	—	—	—	—	—	—
Ragnarsson et al., 2023^14^	7/152	183/1,127	28/239	—	—	218/1,518	143/974	71/528	17/132	24/143	—	117/883
Ailawadi et al., 2024^24^	—	6/188	—	—	—	6/188	—	—	—	—	—	—
Brescia et al., 2024^20^	—	23/417	—	—	—	23/417	—	—	—	—	—	—
Fu et al., 2024 ^11^	26/214	120/868	9/74	34/130	29/110	103/1,176	69/684	73/662	—	—	—	—
Faerber et al., 2024^15^	—	101/1,110	—	—	—	101/1,110	—	—	18/130	43/301	100/964	67/578
Kassab et al., 2024^12^	748/3,442	929/4,852	141/809	211/1,052	967/3,306	1,062/6,849	894/5,464	1,622/7,829	159/913	771/3,928	2,090/10,915	1,836/9,379
Olsthoorn et al., 2024^23^	—	30/227	—	—	—	—	—	—	—	—	—	—

Abbreviations: AV, aortic valve; MV, mitral valve; PPM, permanent pacemaker; TV, tricuspid valve.

Data are reported as PPM *n*/total *N.*

Across the included studies, the reported findings showed substantial variability in the predictors and incidence of PPM implantation after TV surgery. Early work by Cooper et al. reported that nearly one-quarter of patients undergoing TV replacement required PPM implantation.^22^ In a multicenter study, Mar et al. identified prolonged cross-clamp time (>60 min) and concomitant mitral surgery as independent predictors of PPM need, with long-term pacing dependence being more frequent after TV replacement.^13^ Similarly, Kho et al. found that combined mitral–tricuspid operations carried a higher risk of PPM implantation than isolated valve procedures.^6^ Chi et al. observed an overall rise in postoperative pacing rates over time, which was associated with early adverse outcomes.^21^ The randomized trial by Gammie et al. reported that adding tricuspid annuloplasty to degenerative mitral surgery increased the rate of PPM implantation to approximately 14% compared with 2.5% in mitral-only cases.^25^

Nationwide registry data from Ragnarsson et al. confirmed that concomitant ablation and mitral interventions significantly heightened short-term PPM risk, although long-term survival remained unaffected.^14^ Recent multicenter investigations consistently demonstrated that age, baseline ventricular function, and the extent of multiple valve involvement were associated with higher pacing rates.^12,15,20,23,24^ In a large cohort analyzed by Fu et al., the overall incidence of PPM implantation reached 11%, varying by the complexity of concomitant procedures.^11^ Collectively, these studies underscore that PPM implantation remains a frequent postoperative event, particularly in patients undergoing combined valve repairs or replacements.

### Incidence of PPM Implantation Based on Gender Following Different TV Surgeries

**Fig. 4.**
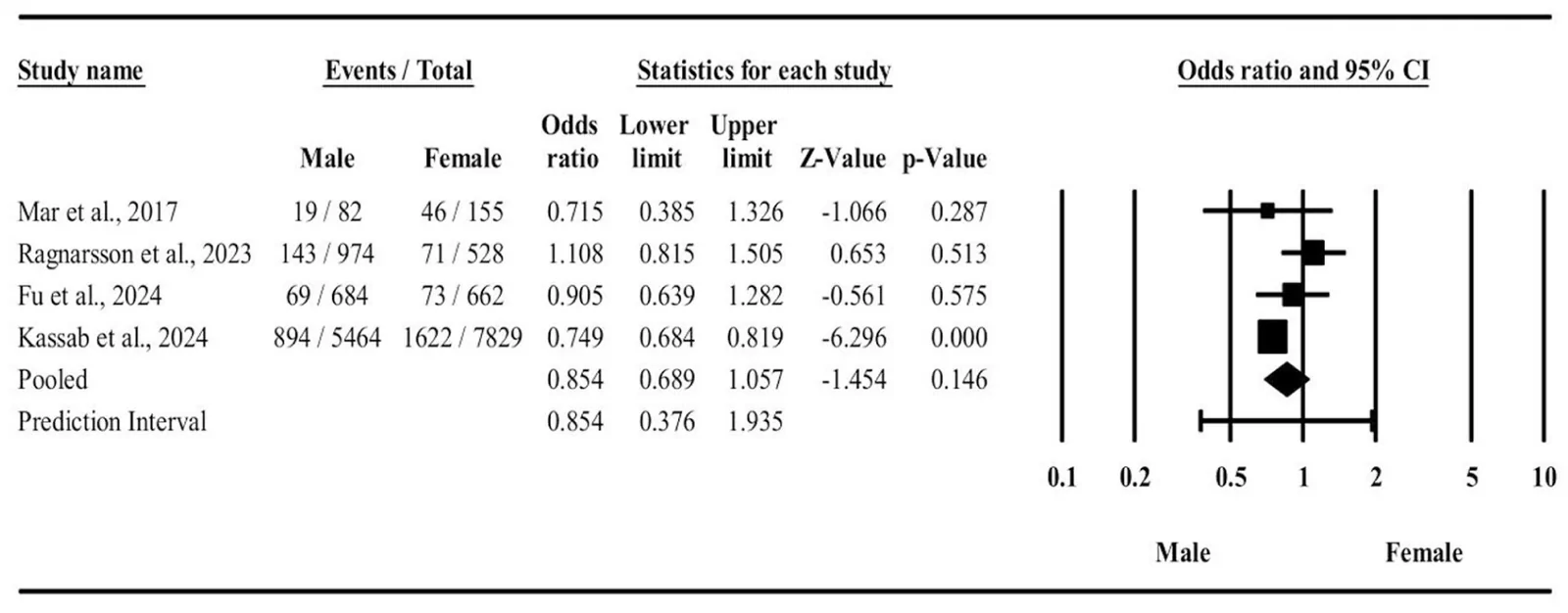
The odds ratio of permanent pacemaker implantation based on gender. CI, confidence interval.

As shown in Figure 4, the OR for the incidence of PPM implantation following various TV surgeries did not differ significantly between genders (OR = 0.85, 95% CI: 0.69 to 1.06, *Z* = −1.45, *P* = 0.146). The incidence of PPM implantation following TV surgeries in male patients was 14.9% (95% CI: 11.8% to 18.5%) and in female patients was 17.6% (95% CI: 12.1% to 24.8%).

### Incidence of PPM Implantation Based on TV Repair or Replacement Surgeries

**Fig. 5.**
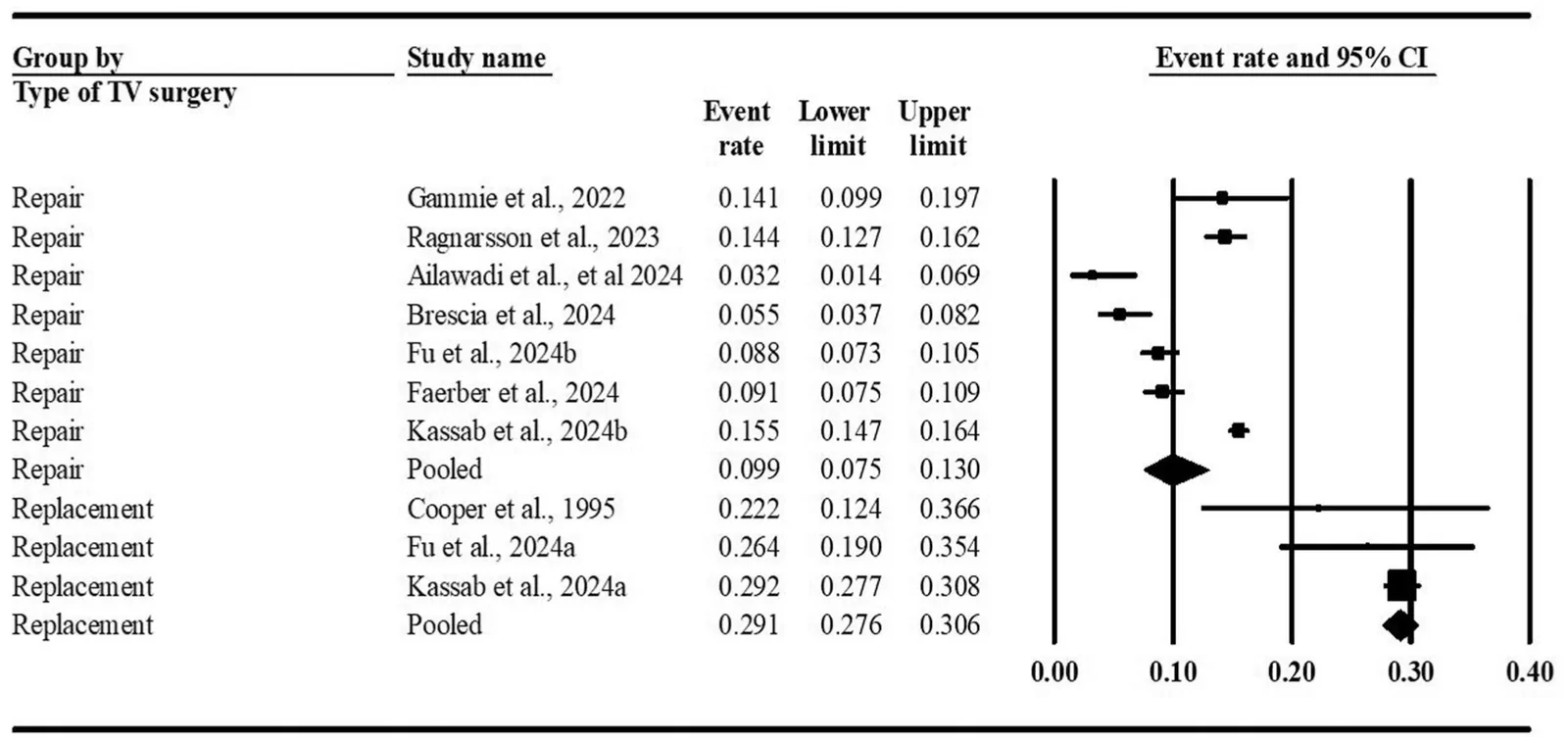
Incidence of permanent pacemaker implantation based on type of TV surgery (repair or replacement). CI, confidence interval; TV, tricuspid valve.

As shown in Figure 5, the incidence of PPM implantation in TV replacement surgery was 29.1% (95% CI: 27.6% to 30.6%), and the incidence of PPM implantation in TV repair surgery was 9.9% (95% CI: 7.5% to 13.0%). Also, the differences between 2 groups was statistically significant (Q = 68.52, *P* < 0.001).

### Incidence of PPM Implantation Following Different TV Surgeries Based on Countries

**Fig. 6.**
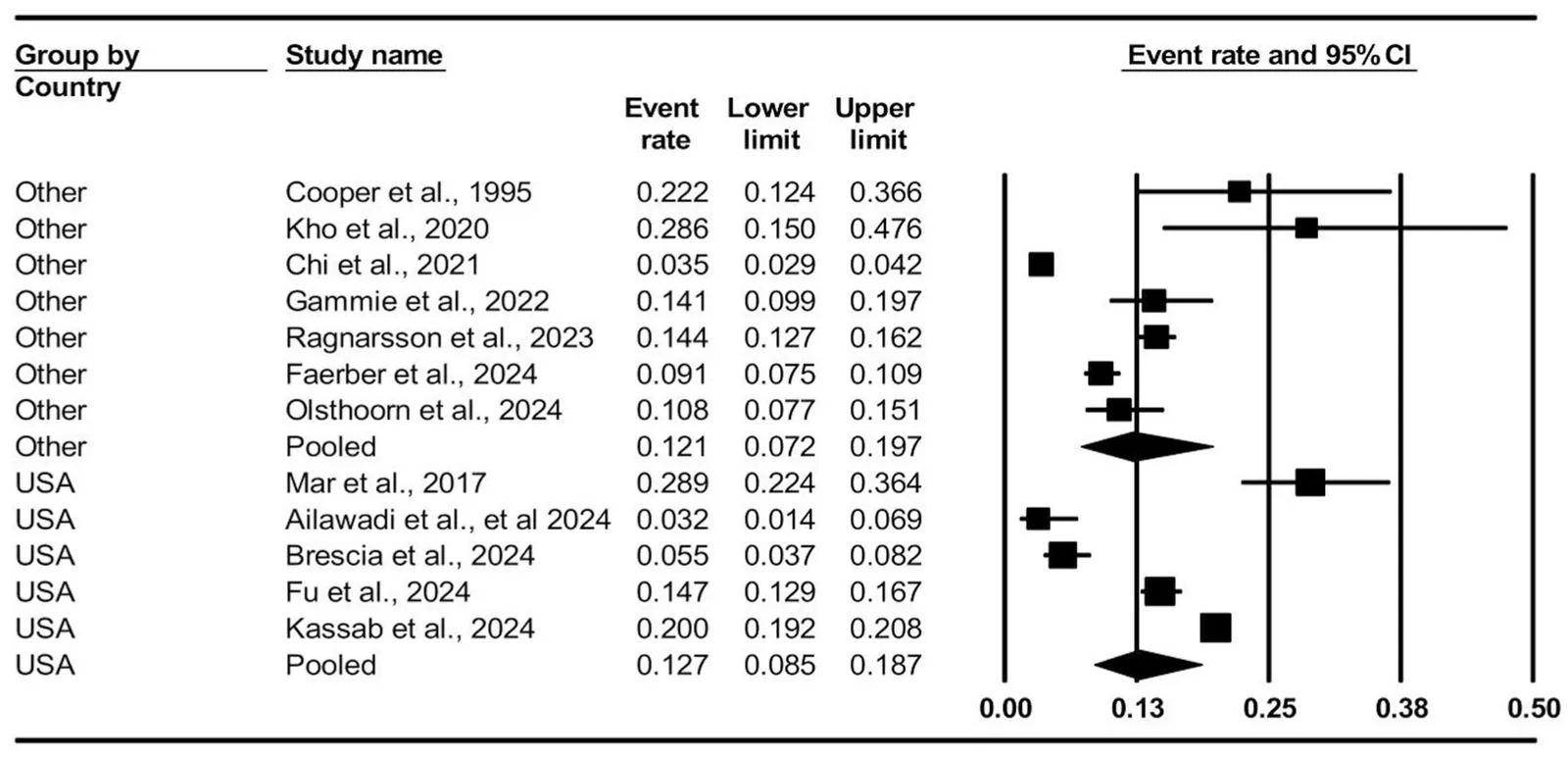
Incidence of permanent pacemaker implantation following different tricuspid valve surgeries based on countries. CI, confidence interval.

As shown in Figure 6, the incidence of PPM implantation in the United States was 12.7% (95% CI: 8.5% to 18.7%), and the incidence of PPM implantation in other countries was 12.1% (95% CI: 7.2% to 19.7%). Also, the differences between the 2 groups were not statistically significant (Q = 0.02, *P* = 0.886).

### Incidence of PPM Implantation Following Different TV Surgeries by Other Risk Factors

As presented in Table 3, the combined incidence of PPM implantation following various TV surgeries in patients with ischemic heart disease, reported in 3 studies, was 25.5% (95% CI: 9.5% to 52.6%, I^2^ = 97.56%, *P* < 0.001). The combined incidence of PPM implantation in patients with diabetes, reported in 4 studies, was 18.3% (95% CI: 14.9% to 22.4%, I^2^ = 60.36%, *P* = 0.052). However, the results of the meta-regression showed that this risk factor had no significant effect on PPM implantation (coefficient = 13.35, 95% CI: −1.86 to 28.55, *P* = 0.085, I^2^ residual = 63.25%). For patients with hypertension, the combined incidence of PPM implantation, reported in 3 studies, was 18.3% (95% CI: 11.3% to 28.2%, I^2^ = 96.48%, *P* < 0.001). In addition, the combined incidence of PPM implantation in patients with heart failure, reported in 4 studies, was 14.7% (95% CI: 10.1% to 20.7%, I^2^ = 95.50%, *P* < 0.001).

**Table 3.** Incidence of PPM Based on Risk Factors.

			95% CI		Heterogeneity			Egger’s regression	
Variable	Number of reports	Pooled incidence	Lower limit	Upper limit	χ^2^	*P* value	I^2^	*t*	*P* value
Ischemic heart disease	3	25.5%	9.5%	52.6%	81.94	<0.001	97.56	0.44	0.736
Diabetes	4	18.3%	14.9%	22.4%	7.57	0.056	60.36	0.43	0.707
Hypertension	3	18.3%	11.3%	28.2%	56.73	<0.001	96.48	0.24	0.849
Heart failure	3	14.7%	10.1%	20.7%	40.41	<0.001	95.05	28.70	0.022

Abbreviations: CI, confidence interval; PPM, permanent pacemaker.

### Incidence Trends of PPM Implantation Following TV Surgeries in Different Years

**Fig. 7.**
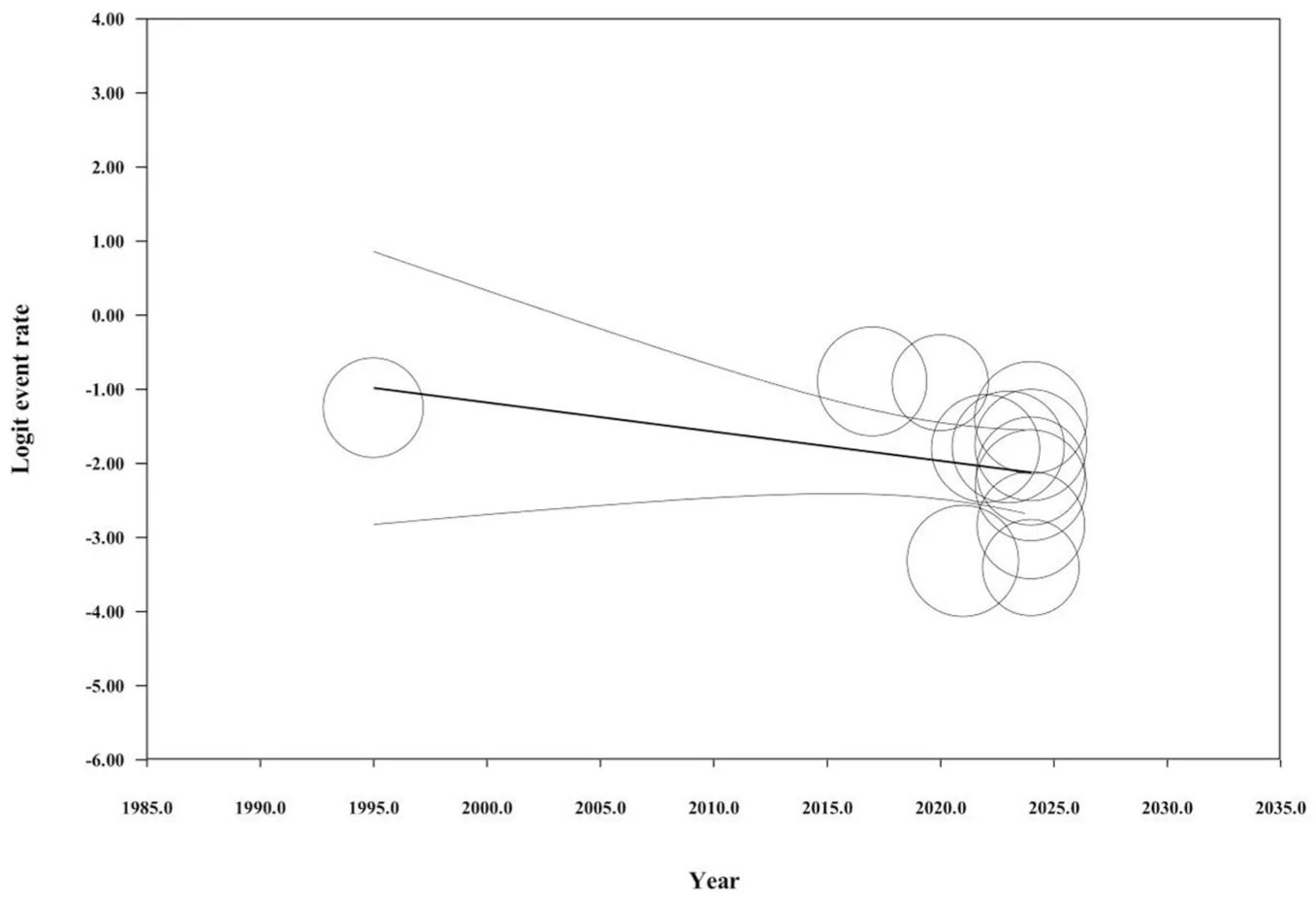
Incidence trends of permanent pacemaker implantation following tricuspid valve surgeries in different years.

The trends of PPM implantations following TV surgeries based on publication year of the article are shown in Figure 7. The results showed that the incidence rate has decreased over the years, but this decrease was not statistically significant (coefficient = −0.04, *Z* = −1.39, *P* = 0.165, I^2^ residual heterogeneity = 97.85%).

### Incidence Trends of PPM Implantation Following TV Surgeries in Different Ages

**Fig. 8.**
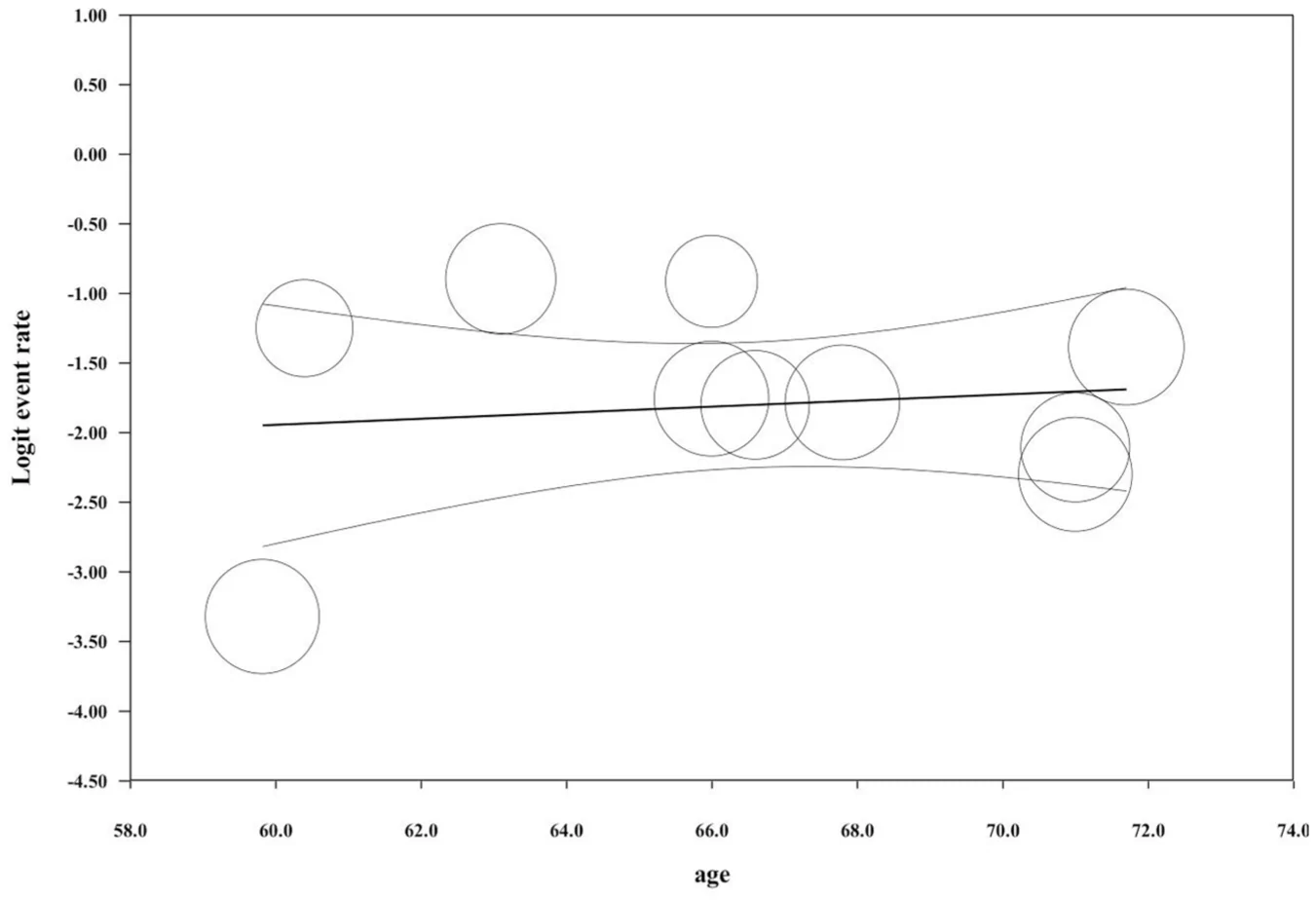
Incidence trends of permanent pacemaker implantation following tricuspid valve surgeries in different ages.

The trends of PPM implantations following TV surgeries based on the age of the patients are shown in Figure 8. The results showed that the incidence rate increased when the age was older, but this increase was not statistically significant (coefficient = 0.02, *Z* = 0.48, *P* = 0.632, I^2^ residual = 88.31%).

### Sensitivity Analysis

**Fig. 9.**
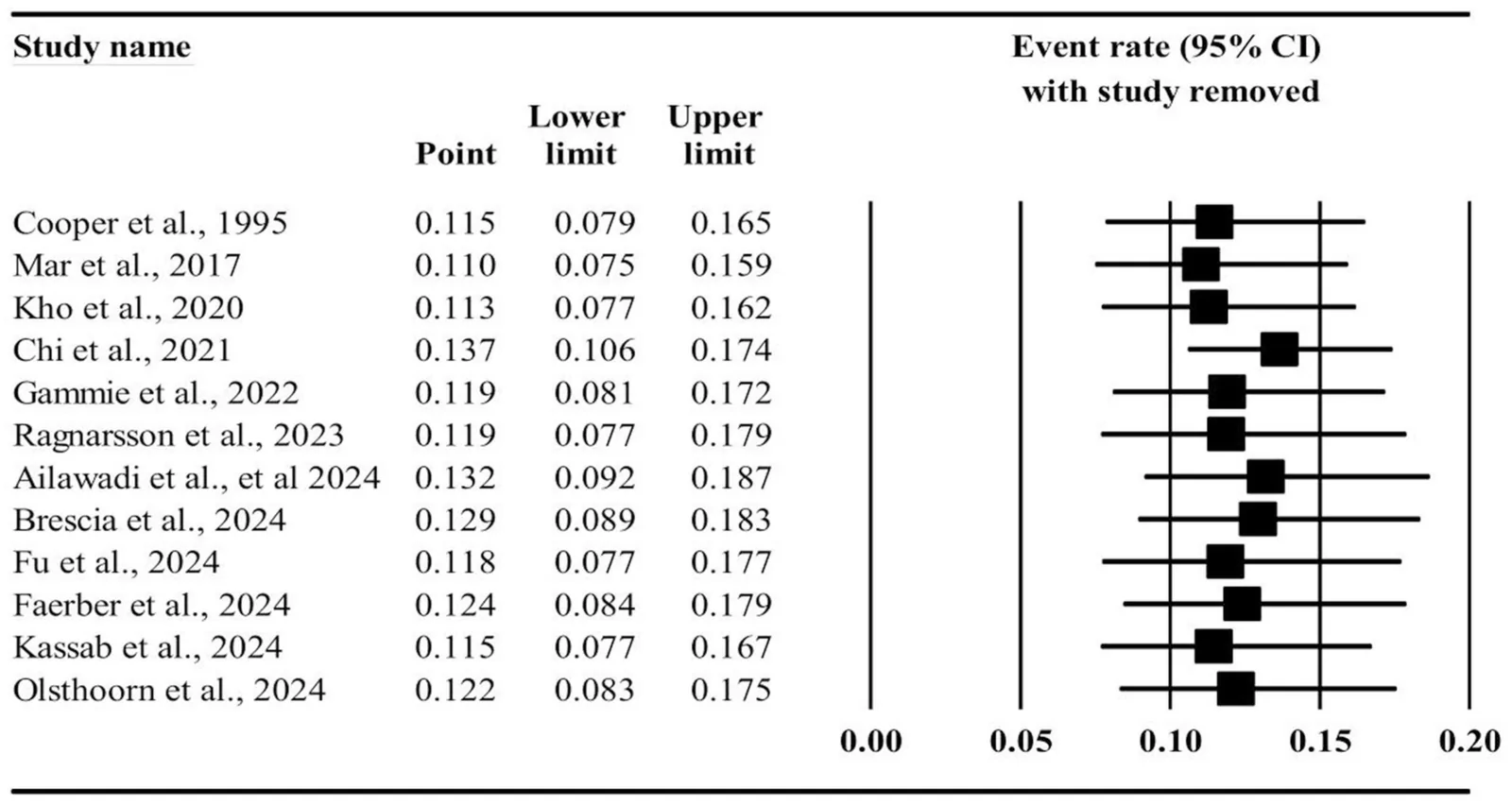
Incidence of permanent pacemaker implantation following tricuspid valve surgeries if 1 study is removed. CI, confidence interval.

As shown in Figure 9, the results of the sensitivity analysis show that the overall event rate (incidence of PPM implantation) was stable across all included studies. No single study had a disproportionate effect on the overall results. The significant *P* values (*P* < 0.001) across all the studies suggest that the findings are robust and not overly influenced by any single study.

### Publication Bias

Publication bias was visually observed in the funnel plots for PPM implantation following TV surgery (Supplemental Fig. 2) and concomitant valve surgeries (TV+MV, TV+AV, and TV+MV+AV; Supplemental Fig. 3, Supplemental Fig. 4). However, the regression tests did not confirm the presence of publication bias for any of these groups, with the following results: TV surgery (*t* = 1.39, *P* = 0.207), TV+MV surgery (*t* = 1.47, *P* = 0.172), TV+AV surgery (*t* = 2.01, *P* = 0.138), and TV+MV+AV surgery (*t* = 0.31, *P* = 0.776).

As indicated in Table 3, the Egger regression test results showed no significant evidence of publication bias in the incidence of PPM implantation following different TV surgeries among patients with ischemic heart disease (*t* = 0.44, *P* = 0.736), diabetes (*t* = 0.43, *P* = 0.707), or hypertension (*t* = 0.24, *P* = 0.849). However, the Egger regression test suggested the presence of publication bias in the incidence of PPM implantation among patients with heart failure (*t* = 28.70, *P* = 0.022).

## Discussion

This systematic review and meta-analysis synthesized the available evidence on the incidence and determinants of PPM implantation following TV surgery. The findings revealed substantial variability in reported incidence rates, largely influenced by the type and complexity of surgery. Patients undergoing concomitant multivalve operations, particularly those involving the MV and AV, demonstrated a significantly higher probability of requiring PPM implantation compared with those undergoing isolated TV procedures.

The incidence of PPM implantation increases markedly when multiple valves are addressed during the same procedure, particularly in TV+MV+AV.^11–13^ This can be attributed to the greater surgical complexity, prolonged cardiopulmonary bypass and cross-clamp times, and more extensive manipulation of cardiac structures adjacent to the conduction system. A New Zealand study found that longer cross-closure times were associated with higher PPM rates, but the independent driving factor was multi-valve surgery rather than time itself.^26^ In a US study, procedural complexity and concurrent surgery were identified as stronger predictors of pacemaker implantation than bypass duration alone.^27^ The anatomical proximity of the atrioventricular node and His bundle to the tricuspid and aortic annuli renders these structures highly susceptible to surgical trauma, explaining the higher pacing rates observed in such cases.^28–30^ The mechanisms underlying conduction system injury during TV surgery are primarily anatomical and procedural. The atrioventricular node is located at the apex of the triangle of Koch, adjacent to the septal leaflet of the TV and the membranous septum, whereas the His bundle penetrates the central fibrous body before running along the interventricular septum beneath the septal annulus. This close spatial relationship places the conduction system within millimeters of the surgical field during annular dissection, suture placement, or prosthetic ring implantation.^31^ Excessive traction on septal sutures, deep bites into the fibrous annulus, or manipulation near the septal commissure can directly injure the atrioventricular node or His bundle, leading to complete heart block or bundle-branch block.^32^ In valve replacement, the risk is further heightened because anchoring sutures and prosthetic seating often involve the membranous septum, where the conduction tissue resides.^33^ In addition, indirect mechanisms such as edema, ischemia from disrupted septal arterial branches, and postoperative inflammation may cause transient or delayed conduction impairment.^34^ These factors increase the likelihood of conduction pathway injury and, consequently, the need for PPM implantation after surgery.

Although the pooled incidence of PPM implantation appeared slightly higher among women than men, this difference did not reach statistical significance. It is therefore likely that sex is not an independent determinant of conduction system disturbance following TV surgery. The small observed variation may reflect sample composition or reporting differences among studies rather than a true biological effect.^11,12,14^ The apparent discrepancies across studies likely represent methodological and population heterogeneity rather than consistent sex-related patterns. Future large-scale studies with stratified sex-based reporting are warranted to clarify these associations.

The present subgroup analysis demonstrated a substantially higher incidence of PPM implantation following TV replacement compared with TV repair, a difference that was statistically significant. This finding aligns with prior reports suggesting that TV replacement poses a greater risk of postoperative conduction disturbances than repair procedures.^13,14,21,24^ The elevated risk can be attributed to the more extensive annular dissection and prosthetic ring implantation required during replacement, which places the atrioventricular conduction axis at higher risk of mechanical injury.^7,8,28^ Conversely, TV repair techniques, especially annuloplasty-based approaches, tend to preserve native anatomic structures and are therefore less disruptive to the conduction system.^14,15^ Both Ragnarsson et al.^14^ and Kassab et al.^12^ reported similar trends, noting that PPM implantation was predominantly associated with valve replacement rather than repair. Collectively, these findings emphasize the importance of preserving the native tricuspid annulus whenever technically feasible to minimize conduction system damage and reduce the postoperative need for permanent pacing.

Several comorbid conditions, including ischemic heart disease, hypertension, diabetes mellitus, and heart failure, were associated with higher rates of PPM implantation following TV surgery. These factors likely increase vulnerability to conduction disturbances through mechanisms such as microvascular ischemia, myocardial fibrosis, and structural remodeling. Ischemic heart disease may compromise the conduction system’s blood supply,^35^ whereas hypertensive and diabetic remodeling contribute to fibrotic changes that alter electrical conduction.^36–38^ Furthermore, chronic heart failure induces adverse chamber dilation and right-sided pressure overload, further predisposing to conduction abnormalities.^39^ Surgeons may minimize trauma by avoiding deep sutures along the septal annulus and limiting traction near the septal commissure, where the atrioventricular node and proximal conduction axis lie near the hinge of the septal leaflet; this is standard practice in tricuspid annuloplasty and is emphasized in contemporary surgical technique descriptions. Using flexible or partial annuloplasty rings helps preserve physiologic annular motion; when combined with avoiding suture bites along the septal annulus, this approach limits mechanical stress in the region of the conduction tissue.^40^ In complex or redo cases, anatomic identification/visualization of the triangle of Koch during repair can further reduce the risk of inadvertent injury to the atrioventricular node, and recent technical reports explicitly describe keeping sutures cranial to Koch’s triangle to avoid atrioventricular block.^41^ In addition, temporary epicardial pacing leads placed at the time of surgery facilitate recognition and management of perioperative conduction instability during weaning from cardiopulmonary bypass.^42^

Postoperatively, close rhythm surveillance, including continuous telemetry and serial electrocardiogram review, together with correction of reversible contributors supports recovery from transient conduction block and can avoid premature PPM implantation.^42^ Major society guidance notes that, when clinically safe, deferring PPM for approximately 5 to 7 days after cardiac surgery is reasonable to allow recovery; contemporary cardiothoracic practice reviews similarly note that many centers delay PPM by 7 to 14 days if the patient is stable because conduction often improves.^42,43^

Although our review focused on structural and procedural predictors, infective endocarditis represents an additional and clinically important risk factor for postoperative conduction disturbances. Endocarditis can lead to tissue destruction, annular abscess formation, or extension of infection to the atrioventricular conduction system, all of which increase the likelihood of requiring permanent pacing after valve surgery.^1,3^ Because most included studies did not separately report endocarditis cases, its specific contribution to PPM risk could not be quantified in this meta-analysis. Future research should isolate this subgroup to clarify the extent of risk and inform surgical decision-making in infective cases.

The underlying AV pathology may also play an important role in the development of postoperative conduction disturbances. In our review, most studies involving concomitant AV procedures likely reflected degenerative aortic stenosis, as this remains the most frequent indication for surgical aortic intervention in contemporary practice.^29,30^ The presence of heavy calcification and fibrosis surrounding the aortic annulus in degenerative disease increases the risk of mechanical trauma to the atrioventricular conduction system, which lies in close proximity to the aortic root.^8,28,37^ In contrast, rheumatic aortic disease, although less prevalent in recent cohorts, can involve diffuse fibrotic extension that may also influence conduction outcomes.^35,36^ Large multicenter studies have demonstrated that combined multivalve procedures, particularly those involving the AV and TV, are associated with longer operative times and a higher incidence of postoperative pacemaker implantation.^29,30,44^ Because most of the included studies did not specify the precise etiology of AV disease, we were unable to perform stratified analysis. Future investigations should report these distinctions systematically to better determine how degenerative versus rheumatic mechanisms affect conduction system injury and pacing risk.

The presence of these additional health issues underscores the importance of thorough preoperative assessments and tailored risk management plans. By employing specialized surgical methods and closely monitoring patients with these conditions, we can potentially lower the chances of needing PPM implantation after TV procedures.

### Heterogeneity Considerations

The high degree of heterogeneity observed across studies (I^2^ > 90% in several analyses) reflects substantial clinical and methodological variability. This variation likely arises from differences in baseline patient profiles, comorbidities, surgical techniques (repair vs replacement), concomitant valve interventions, and institutional implantation criteria. Although such heterogeneity limits the precision of pooled estimates, the application of a random-effects model, supplemented with meta-regression and sensitivity analyses, allows for cautious yet meaningful synthesis of available evidence. Accordingly, the presented pooled incidence rates should be interpreted as representative trends rather than absolute estimates applicable to any single institution or patient population.

### Limitations

This study has several limitations that should be acknowledged. First, substantial clinical and methodological heterogeneity was observed across the included studies, as reflected by the high I^2^ values. This variability likely stems from differences in patient characteristics, comorbid conditions, surgical techniques, concomitant procedures, follow-up durations, and institutional thresholds for PPM implantation. Second, although random-effects modeling, meta-regression, and sensitivity analyses were used to address this heterogeneity, these methods cannot fully mitigate its influence on pooled estimates; therefore, the reported incidence rates should be interpreted as indicative trends rather than absolute values. Third, most studies did not distinguish between early (in-hospital or ≤30 days) and late (>30 days) PPM implantation. Consequently, the pooled incidence reflects overall implantations without temporal stratification, which may overestimate the true rate of long-term pacing dependency. Fourth, the predominance of observational study designs introduces potential sources of selection bias, unmeasured confounding, and variability in reporting quality. Moreover, incomplete documentation of key operative details across studies limited the feasibility of more granular subgroup analyses. In addition, few studies provided detailed information regarding the etiology of concomitant MV or TV disease (degenerative vs rheumatic), restricting the assessment of how the underlying pathology influences PPM risk. Data concerning infective endocarditis were similarly sparse and inconsistently reported, precluding a dedicated analysis for this clinically significant subgroup. Finally, the lack of consistent reporting on whether tricuspid pathology was functional or organic limited our ability to explore differences in postoperative conduction outcomes between these categories.

### Recommendations for Future Research

Future research should prioritize prospective studies aimed at clarifying the causative factors leading to PPM implantation and evaluating its long-term effects on patient outcomes. Also, future research should clearly report the timing and persistence of pacing dependency to better guide clinical decision-making regarding when permanent devices are truly warranted. Future research should report this distinction more systematically, as the underlying pathology could meaningfully influence surgical strategy and postoperative conduction outcomes. In addition, investigations into alternative surgical techniques or perioperative management strategies that could reduce the incidence of PPM implantation would be highly valuable. Such studies have the potential to significantly enhance our understanding of optimal management approaches for patients undergoing TV surgery.

## Conclusions

This systematic review and meta-analysis provides a comprehensive overview of PPM implantation after TV surgery. Our findings reveal that PPM is a common complication, especially in patients undergoing complex multivalve procedures. The present results call attention to just how pivotal it is to have individualized preoperative risk assessments and to prepare clear counseling to patients, especially when they undergo concomitant valve surgery. Although we have attempted to adjust for differences in study designs, the variability in reporting makes it difficult to get precise pooled estimates. There is a need for future prospective studies that use standardized definitions, consistent surgical reporting, and long-term follow-up to boost clinical guidance. Such initiatives will be vital for improving risk prediction and designing strategies to reduce the risk of conduction system injury.

## Supplemental Material

sj-docx-1-inv-10.1177_15569845261455899 – Supplemental material for Incidence of Permanent Pacemaker Implantation Following Tricuspid Valve Surgeries: A Systematic Review and Meta-Analysis Supplemental material, sj-docx-1-inv-10.1177_15569845261455899 for Incidence of Permanent Pacemaker Implantation Following Tricuspid Valve Surgeries: A Systematic Review and Meta-Analysis by Seyed Shervin Shafiei, Saeid Hosseini, Samuel Heuts, Abolfazl Hosseinnataj and Peyman Sardari Nia in Innovations
