# Supplementary material for: Incidence of Permanent Pacemaker Implantation Following Tricuspid Valve Surgeries: A Systematic Review and Meta-Analysis
Source: Innovations (Phila). 2026 Jun 16;21(3):217–30. doi: 10.1177/15569845261455899 (PMC13534769; doi:10.1177/15569845261455899)
Supplement: Supplementary file 1 [file sj-docx-1-inv-10.1177_15569845261455899.docx]

**Incidence of Permanent Pacemaker Implantation Following** **Tricuspid Valve Surgeries: A Systematic Review and Meta-Analysis**

**Supplemental Figure 1.** Risk of bias assessment of included studies using ROBINS-I (observational cohorts) and RoB-2 (randomized trial).

**Supplemental Figure 2.** Funnel plot of permanent pacemaker implantation following overall tricuspid valve surgeries.

**Supplemental Figure 3.** Funnel plot of permanent pacemaker implantation following tricuspid valve surgery (left) and tricuspid valve + mitral valve surgery (right).

**Supplemental Figure 4.** Funnel plot of permanent pacemaker implantation following tricuspid valve + aortic valve surgery (left) and tricuspid valve + mitral valve + aortic valve surgery (right).
